# Increase in HIV viral suppression in KwaZulu-Natal, South Africa: Community-based cross sectional surveys 2018 and 2013. What remains to be done?

**DOI:** 10.1371/journal.pone.0265488

**Published:** 2022-03-24

**Authors:** Nolwenn Conan, Erica Simons, Menard L. Chihana, Liesbet Ohler, Ellie FordKamara, Mduduzi Mbatha, Gilles vanCutsem, Helena Huerga

**Affiliations:** 1 Epicentre, Paris, France; 2 Médecins sans Frontières, Eshowe, South Africa; 3 Department of Health of South Africa, South Africa; 4 Médecins sans Frontières, Southern Africa Medical Unit, Cape Town, South Africa; 5 Centre for Infectious Disease Epidemiology and Research, University of Cape Town, Cape Town, South Africa; Centers for Disease Control and Prevention, UNITED STATES

## Abstract

**Introduction:**

High coverage of antiretroviral therapy (ART) in people living with HIV (PLHIV) increases viral suppression at population level and may reduce incidence. Médecins sans Frontières, in collaboration with the South African Department of Health, has been working in Eshowe/Mbongolwane (KwaZulu Natal) since 2011 to increase access to quality HIV services. Five years after an initial survey, we conducted a second survey to measure progress in HIV diagnosis and viral suppression and to identify remaining gaps.

**Methods:**

A cross-sectional, population-based, stratified two-stage cluster survey was implemented in 2018, using the same design as in 2013. Consenting participants aged 15–59 years were interviewed and tested for HIV at home. Those HIV-positive were tested for HIV viral load (viral suppression defined as <1000 copies/mL).

**Results:**

Overall, 3,278 individuals were included. The proportion of HIV-positive participants virally suppressed was 83.8% in 2018 compared to 57.1% in 2013 (p<0.001), with increases in all subpopulations. The largest gap remained in men aged 15–29 years, among whom viral suppression was 51.5%. Nevertheless, of the total unsuppressed participants, 60.3% were women, and 57.4% were individuals aged 30–59 years. Between 2013 and 2018, HIV-positive status awareness progressed from 75.2% to 89.9% and ART coverage among those aware from 70.4% to 93.8%, respectively. Among those on ART, 94.5% were virally suppressed in 2018.

**Conclusions:**

Viral suppression improved significantly from 2013 to 2018, in all age and gender groups of PLHIV. However, almost half of HIV-positive young men remained unsuppressed, while the majority of virally unsuppressed PLHIV were women and older adults. To continue lowering HIV transmission, specific strategies are needed to increase viral suppression in those groups.

## Introduction

In the last decade, progress in HIV prevention research has transformed the scope of HIV programs and raised expectations that very high coverage of antiretroviral therapy (ART), increasing viral suppression at population level, may reduce incidence. Cumulative evidence has demonstrated that HIV-positive individuals on ART who achieve and maintain an undetectable HIV-ribonucleic acid (RNA) viral load (VL) do not sexually transmit the virus to others [1–3]. Correspondingly, mathematical models suggested that HIV incidence reduction would be feasible if HIV-positive persons had early access to diagnosis and treatment [4,5]. In 2016, the Prevention Access Campaign launched the Undetectable = Untransmissible (U = U) slogan to promote these findings [6,7].

South Africa aimed to achieve at least 73% of viral load suppression of all persons living with HIV (PLHIV) by 2020 [8,9]. Despite a major decrease in HIV incidence and a reduction in AIDS-related mortality since 2010 [10], South Africa’s HIV prevalence of 20.6% is one of the highest in the world and the prevalence of 27.0% in KwaZulu-Natal (KZN) province is the highest in the country [11]. In 2018, an estimated 7.7 million South Africans were still living with HIV, among whom 62% were accessing ART [10]. The country adopted the “universal test and treat” (UTT) strategy in 2015 and started implementation in June 2016 [12,13].

In 2011, Médecins sans Frontières (MSF) in collaboration with the Department of Health (DoH) opened a project to support the HIV/AIDS program in 14 administrative wards of Umlalazi Municipality (KZN Province), which has an estimated population of 114,490 [14]. The project included prevention activities, HIV counselling and testing (HCT) door-to-door and at fixed community testing sites, support for linkage to care and early ART initiation. In addition, the project aimed to improve retention in care and viral suppression by supporting standard clinic-based care, ART initiation and adherence counselling, differentiated models of care, mentoring on implementation of the national adherence and clinical guidelines, including scale-up of viral load monitoring, and management of treatment failure.

In 2013, a population-based survey in the area assessed parameters of the HIV epidemic [15]. The findings of that survey helped to adapt the strategy in the sub-district. In a context where UTT had been implemented since 2016 and following five years of implementation activities, it was expected that progress towards United Nations Programme on HIV/AIDS (UNAIDS) 90-90-90 targets, and consequently viral suppression in PLHIV, would have improved in this setting, leading to decreased HIV transmission and a reduction in HIV incidence. Five years following the first survey in 2013, we conducted a similar survey to measure progress in HIV diagnosis and viral suppression and to identify remaining gaps.

## Materials and methods

### Design and population

We conducted a cross-sectional population-based survey between August and December 2018, in the 14 administrative wards of Umlalazi Municipality (KZN) where MSF operated, using two-stage cluster sampling. The sample size was calculated to estimate a difference of 7% (with a precision of +/-6%) in the viral load suppression among men between 2013 and 2018, as men were less numerous than women. In total 2,167 households were planned to be visited in 2018 to recruit 2,492 men. In the first stage, we selected 87 small area layers (SALs) using probability proportional to population size. SALs were selected with the sampling frame based on the South African Population and Housing Census, provided by Statistics South Africa [14]. In the second stage, twenty-five dwellings were randomly selected in each SAL, using point-based spatial sampling from a list of points, making the sample selection self-weighted. Vacant, destroyed or non-located dwellings were replaced using a list of reserve points. The same design was used in 2013 [15].

### Interviews and laboratory procedures

All adults aged more than 15 years living in the selected households were eligible for the survey and invited to participate. Recruitment of survey participants occurred among residents of the survey area and visitors who had spent at least the previous night in the survey area. The survey teams visited the selected dwellings on different days, with support elicited from household members or neighbours to arrange a return visit if a household member was absent. Occupants who were not located on a third visit or who refused to participate were not replaced. A face-to-face questionnaire was administrated to eligible participants to collect information on demographic characteristics and HIV testing habits. Individuals testing HIV-positive during the survey were asked to self-report the date of their positive HIV test (if previously tested) as well as ART intake and start date, which was verified with the health book. Using a serial testing algorithm, HIV testing was proposed to all survey participants, as in 2013 [15], irrespective of knowledge of HIV status or current ART use, at their residence with an HIV rapid test using whole blood obtained by finger-prick. The initial screening used the Determine Rapid HIV-1/2 Antibody test (Abbott Laboratories, Abbott Park, IL, USA), followed, if positive, by the Unigold Rapid HIV Test (Trinity Biotech PLC, Bray, Ireland) for confirmation of results. Both tests have shown a good sensitivity and specificity in different settings, including South Africa [16,17]. Participants positive on both tests were considered positive. Those with discordant results (Determine positive, Unigold negative) had a third “tiebreaker” test using a Western Blot platform (Bio-Rad, USA) to confirm the HIV status. A quality control of the tests occurred through serological testing of 10% of samples tested HIV-positive with rapid test. Pre- and post-counselling was provided to participants, according to the national HCT guidelines [13]. The survey teams collected venous blood samples from participants who tested HIV-positive to perform additional laboratory-based tests. HIV-RNA VL was quantified on plasma HIV-RNA Polymerase chain reaction (Abbott RealTime HIV-1 platform (m2000sp), USA) with a limit of detection of 40 copies/mL in 2018 [18]. In the 2013 survey, NucliSens EasyQ HIV- 1v2.0 assay from Biomerieux had been used (limit of detection of 20 copies/mL). As it was performed in the 2013 survey [15], in 2018 participants who were willing to participate in the survey but not to receive the HIV test on site (and therefore to know their HIV test results), had blood collected in order for HIV and HIV-RNA VL tests to be conducted at the laboratory.

### Definitions

HIV diagnosis and care (UNAIDS 90-90-90 targets): HIV-positive status awareness was defined as at least one HIV diagnosis prior to the survey (report of at least one positive HIV test). Being on ART at the time of the survey was determined by documentation from individual health books. ART coverage was calculated among individuals who were aware of their HIV-positive status. In 2013, ART coverage was established from a positive blood ART detection test [19] and participants with a missing ART blood detection were classified according to self-reported intake. Viral suppression was defined as HIV-RNA VL below 1,000 copies/mL in both 2013 and 2018 surveys. We estimated the proportion of all HIV-positive individuals virally suppressed and the proportion of those on ART virally suppressed.

### Data collection and analysis

Data was captured from paper-based questionnaires, laboratory information management systems and registers according to the standard procedures of each laboratory. Questionnaires were pre-tested prior to the studies’ launches. Data was double entered into EpiData 3.1 (EpiData Association, Odense, Denmark) and statistical analyses were performed with STATA 14 and 15 (StataCorp, College station, Texas, USA). Descriptive analyses, weighted to account for the selection probability of the cluster sampling procedure, were performed. Outcomes were stratified by sex and age groups (15 to 29 years, 30 to 59 years) and reported with corresponding 95% confidence interval (CI). Categorical variables were compared using Pearson’s chi-square or Fisher’s exact test, as appropriate. P-values below 0.05 were considered statistically significant. Proportions with corresponding 95% CI were calculated by excluding any missing values from the denominator.

In 2013, all participants living in the selected household and aged 15 to 59 years were eligible and were invited to participate [15] while in 2018, the survey targeted individuals aged 15 years and older. In order to allow comparisons with the 2013 results, the 2018 analyses reported here included participants 15–59 years of age only.

### Ethical approvals

Ethical approval was received from the University of Cape Town Human Research Ethics Committee (HREC reference: 320/2018), the Provincial Health Research Unit of the KwaZulu Natal Department of Health (NHRD 2018 Ref: KZ_201807_26) and the Médecins Sans Frontières Ethics Review Board (Reference: ID1842). Approvals included the cut off of 15 years of age in seeking guardian consent.

Prior to participation, participants provided written informed consent (in English or isiZulu). Although minors aged 15–17 years did not need guardian consent to participate in the survey, they were advised to disclose their status, regardless of result, to their parent/guardian.

## Results

### Socio-demographic characteristics and HIV prevalence

In 2018, 2,170/2,450 (88.6%) households were eligible for the survey. The inclusion rate among eligible households was 82.7% (1,795/2,170). Of 4,109 eligible individuals, 3,286 (80.0%) were included. Inclusion rate was higher among women than men: 84.7% versus 72.2% (p<0.001). Median age was 30 years [IQR 20–44] and 2,165 participants (65.9%) were women (**Table 1**). Sociodemographic characteristics of participants were similar in 2013 and 2018 (**Table 1**). Most participants were never married, had secondary school education and were residents of Eshowe/Mbongolwane for more than ten years. The proportion of persons who reported being unemployed increased between 2013 and 2018.

**Table 1:** Selected sociodemographic characteristics among men and women aged 15–59, 2013 and 2018 surveys.

	Women		Men		Total	
	2013	2018	2013	2018	2013	2018
	n (%)	n (%)	n (%)	n (%)	n (%)	n (%)
**Age group (years)**	3,518	2,165	2,131	1,121	5,649	3,286
15–19	774 (22.0)	381 (17.6)	679 (31.9)	345 (30.8)	1,453 (25.7)	726 (22.1)
20–24	623 (17.7)	345 (15.9)	436 (20.5)	190 (17.0)	1,059 (18.8)	535 (16.3)
25–29	497 (14.1)	236 (10.9)	295 (13.8)	124 (11.1)	792 (14.0)	360 (11.0)
30–34	306 (8.7)	259 (12.0)	180 (8.5)	96 (8.6)	486 (8.6)	355 (10.8)
35–39	283 (8.0)	184 (8.5)	134 (6.3)	94 (8.4)	417 (7.4)	278 (8.5)
40–44	251 (7.1)	171 (7.9)	117 (5.5)	81 (7.2)	368 (6.5)	252 (7.7)
45–49	259 (7.4)	166 (7.7)	93 (5.5)	67 (6.0)	352 (6.2)	233 (7.1)
50–54	282 (8.0)	201 (9.3)	101 (4.7)	66 (5.9)	383 (6.8)	267 (8.1)
55–59	243 (6.9)	222 (10.3)	96 (4.5)	58 (5.2)	339 (6.0)	280 (8.5)
**Marital Status (n)**	3,515	2,163	2,129	1,120	5,644	3,284
Never Married	2,448 (69.6)	1,441 (66.6)	1,786 (83.9)	921 (82.2)	4,234 (75.0)	2,362 (71.9)
Married/Living Together	905 (25.8)	547 (25.3)	294 (13.8)	176 (15.8)	1,199 (21.2)	724 (22.1)
Divorced/Separated	65 (1.9)	33 (1.5)	39 (1.8)	16 (1.4)	104 (1.8)	49 (1.5)
Widowed	97 (2.8)	142(6.6)	10 (0.5)	7 (0.6)	107 (1.9)	149 (4.5)
**Education (n)**	3,518	2,164	2,130	1,121	5,648	3,286
No schooling	319 (9.1)	204 (9.4)	112 (5.3)	53 (4.7)	431 (7.6)	257 (7.8)
Primary	1,448 (41.2)	845 (39.0)	963 (45.2)	493 (44.0)	2,411 (42.7)	1,338 (40.7)
Secondary	1,625 (46.2)	1,018 (47.0)	988 (46.4)	551 (49.2)	2,613 (46.3)	1,569 (47.8)
Tertiary	126 (3.6)	97 (4.5)	67 (3.2)	24 (2.1)	193 (3.4)	121 (3.7)
**Occupation (n)**	3,518	2,165	2,131	1,120	5,649	3,286
Farmer, Forestry	176 (5.0)	37 (1.7)	141 (6.6)	22 (2.0)	317 (5.6)	59 (1.8)
Salaried employment	495 (22.9)	252 (11.6)	426 (38.0)	177 (15.8)	921 (28.0)	429 (13.1)
Student	876 (24.9)	391 (18.1)	756 (35.5)	360 (32.1)	1,632 (28.9)	751 (22.9)
Housewife/husband	439 (12.5)	193 (8.9)	26 (1.2)	16 (1.4)	465 (8.2)	209 (6.4)
None	1,418 (40.3)	1,216 (56.2)	631 (29.6)	477 (42.6)	2,049 (36.3)	1,693 (51.5)
Other	114 (3.2)	76 (3.5)	151 (7.1)	68 (6.1)	265 (4.7)	144 (4.4)
**Moved residence in 10 years (n)**	3,518	2,126	2,131	1,102	5,649	3,286
Yes	457 (13.0)	206 (9.7)	304 (14.3)	120 (10.9)	761 (13.5)	326 (10.1)
No	3,061 (87.0)	1,920 (90.3)	1,827 (85.7)	982 (89.1)	4,888 (86.5)	2,902 (89.9)

**Fig 1:**
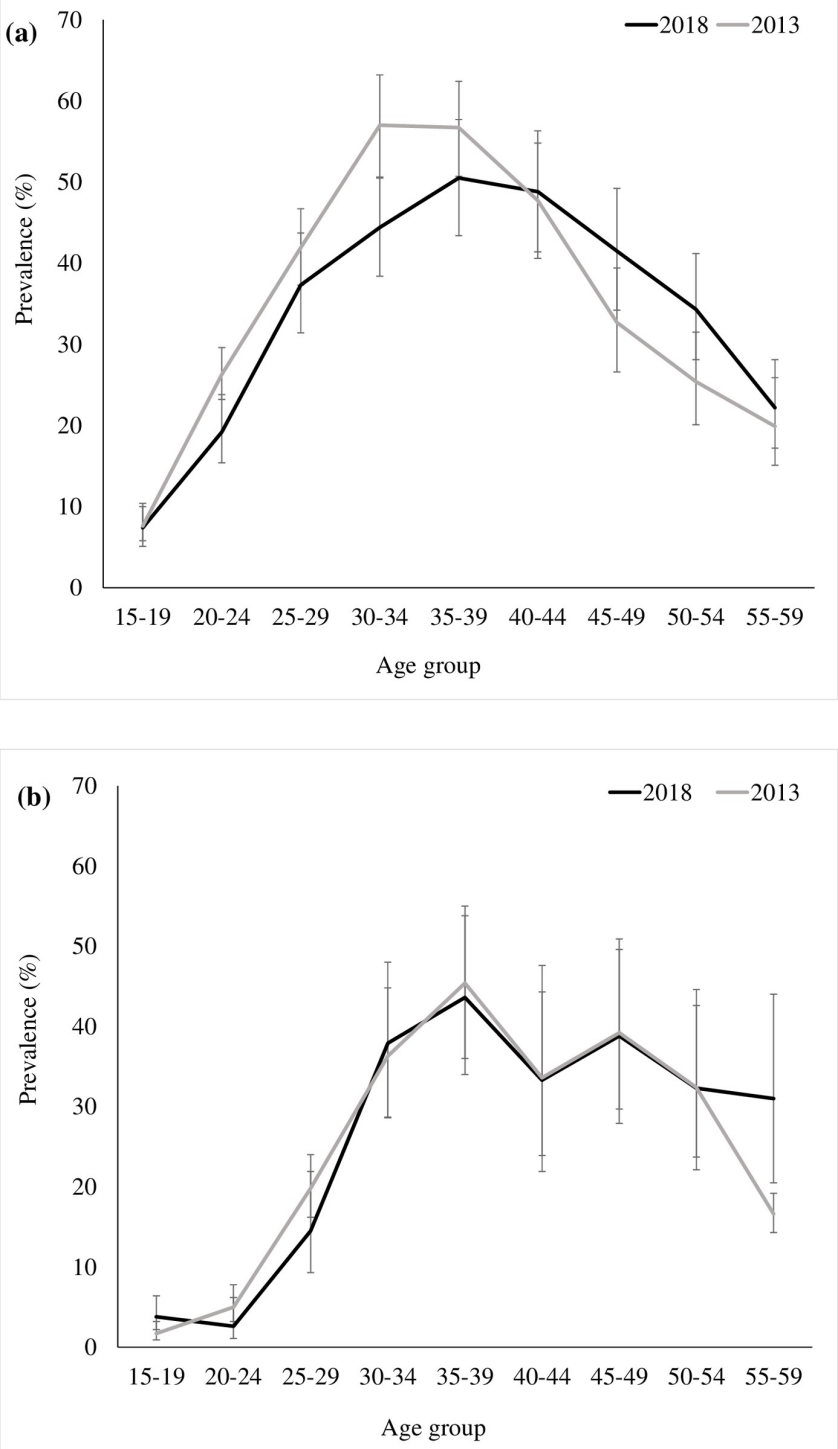
HIV prevalence by five-year age group among women (a) and among men (b), KwaZulu Natal, South Africa, 2013 and 2018.

In total, 862 participants were HIV-positive in 2018. The overall weighted 2018 HIV prevalence was 26.4% (comparable to 25.2% in 2013; p = 0.23). HIV prevalence was higher in women than in men, 30.5% and 18.4%, respectively (p<0.001). HIV prevalence stratified by sex was not statistically different in 2013 or 2018 (**Fig 1**).

### Viral suppression

The proportion of HIV-positive individuals virally suppressed was 83.8% (95%CI: 81.1–86.1) in 2018, a 1.5-fold increase compared to 2013 (57.1%, 95%CI: 54.4–59.6) (**Table 2**). Among women, viral suppression increased from 60.0% in 2013 to 87.2% in 2018 (p<0.001) and among men, from 47.7% to 72.9% (p<0.001). Although younger HIV-positive individuals aged 15–29 years experienced a greater improvement in viral load suppression compared to older individuals, with an increase of about two-fold (p<0.001) between 2013 and 2018, the largest gap in 2018 remained in young men, of whom 51.5% (95CI: 34.8–67.9) were virologically suppressed.

**Table 2:** Viral load supression (VL<1,000 copies/ml) among all hiv-positive participants, by sex and age group, 2013 and 2018 surveys.

	15–29 years				30–59 years				All HIV-positive			
Year	2013		2018		2013		2018		2013		2018	
	n/N	% (95%CI)	n/N	% (95%CI)	n/N	% (95%CI)	n/N	% (95%CI)	n/N	% (95%CI)	n/N	% (95%CI)
**Overall**	203/496	40.9 (36.7–45.3)	147/205	71.7 (65.2–77.5)	593/ 899	66.0 (62.8–69.0)	554/632	87.7 (84.9–90.0)	796/1,395	57.1 (54.4–59.6)	701/837	83.8 (81.1–86.1)
**Women**	182/411	44.3 (39.5–49.1)	130/172	75.6 (68.6–81.4)	456/ 653	69.8 (66.2–73.2)	426/466	91.4 (88.5–93.7)	638/1,064	60.0 (57.0–62.9)	556/638	87.2 (84.3–89.5)
**Men**	21/85	24.7 (16.7–35.0)	17/33	51.5 (34.8–67.9)	137/246	55.7 49.4–61.8)	128/166	77.1 (70.0–82.9)	158/331	47.7 (42.4–53.1)	145/199	72.9 (66.3–78.6)

**Fig 2:**
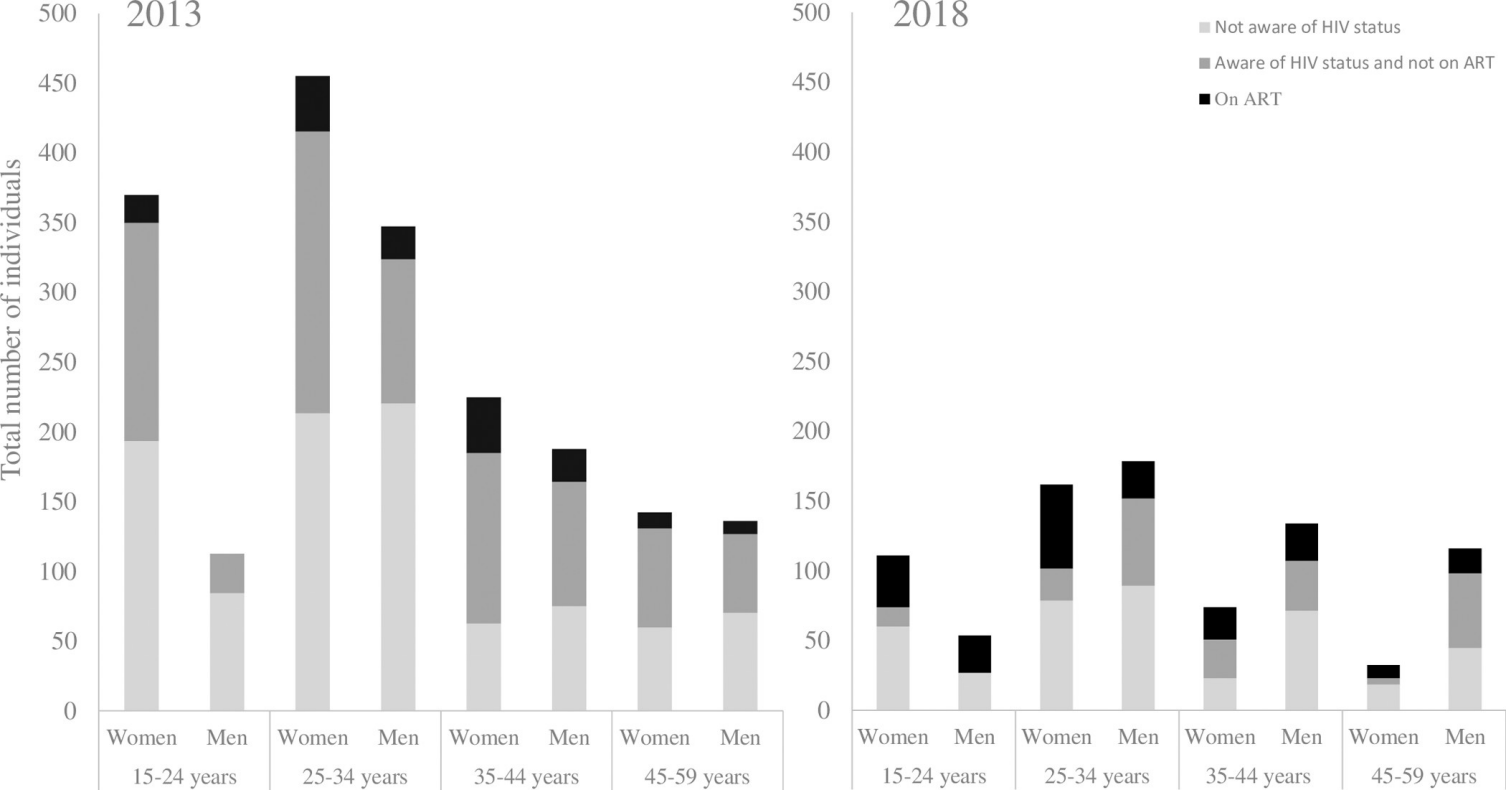
HIV-positive participants with viral load≥1,000 copies/mL, stratified by sex and age according to their HIV status awareness and antiretroviral treatment status, and adjusted to a surveyed population of 10,000 persons, KZN, South Africa, 2013 and 2018.

In 2018, the majority of unsuppressed participants were women (60.3%) and individuals aged 30 years and older (57.4%). Among the unsuppressed, no statistical difference was observed between the two surveys in the proportion of participants unaware of their HIV-positive status (48.1% in 2013 versus 47.8% in 2018, p = 0.95); while the proportion aware of their status and not on ART decreased between 2013 and 2018 (43.2% versus 23.5% respectively, p<0.001) (**Fig 2**). To permit direct comparison between the two surveys, results in **Fig 2** were standardized to a study population of 10,000. In 2018, 90.8% (59/65) of the unsuppressed who were unaware of their HIV-positive status reported that they had received at least one HIV test prior to the survey, and 50.8% (95%CI: 37.5–64.1) (30/59) were tested in the year prior to the survey.

### Achievement of the 90-90-90 targets

The project reached the UNAIDS 90-90-90 targets in 2018, with 89.9% of PLHIV aware of their status, 93.8% of those aware of their status on ART, and 94.5% of those on ART virally suppressed. The three “90s” improved compared to 2013: 75%-70%-93% (p<0.001 for the first two proportions; p = 0.28 for the last one). Overall, HIV diagnosis and care improved in all sex and age categories compared to 2013 (**Table 3**).

**Table 3:** UNAIDS 90-90-90 target results, stratified by sex and age group, 2013 and 2018 surveys.

	Women				Men				15–29				30–59				Overall			
Years	2013		2018		2013		2018		2013		2018		2013		2018		2013		2018	
	n/N	%	n/N	%	n/N	%	n/N	535%	n/N	%	n/N	%	n/N	%	n/N	%	n/N	%	n/N	%
		(95%CI)		(95%CI)		(95%CI)		(95%CI)		(95%CI)		(95%CI)		(95%CI)		(95%CI)		(95%CI)		(95%CI)
**Status awareness among all HIV-positive**	839/1,080	77.7	605/657	92.1	226/336	67.3	170/205	82.9	315/507	62.1	177/217	81.6	750/909	82.5	598/645	92.7	1,065/1,416	75.2	775/862	89.9
**(First 90)**		75.1–80.1		89.8–93.2		62.1–72.1		77.1–87.5		57.8–66.3		75.8–86.2		79.9–84.9		90.4–94.5		73.0–77.4		87.7–91.8
**On ART among those aware**	589/834	70.6	576/602	95.7	152/219	69.4	146/168	86.9	181/314	57.6	160/176	90.9	560/739	75.8	562/594	94.6	741/1,053	70.4	722/770	93.8
**(Second 90)**		67.4–73.6		93.7–97.1		63.0–75.2		81.0–91.2		52.1–63.0		85.7–94.4		72.5–78.8		92.5–96.2		67.5–73.1		91.8–95.3
**Virologically suppressed among those on ART**	549/588	93.4	536/564	95.0	140/152	92.1	132/143	92.3	161/181	89	135/152	88.8	528/559	94.5	533/555	96	689/740	93.1	668/707	94.5
**(Third 90)**		91.0–95.1		92.9–96.6		86.6–95.5		86.6–95.7		83.5–92.8		82.7–92.9		92.2–96.2		94.1–97.4		91.0–94.7		92.5–96.0

Stratifying by sex, all three 90s were surpassed in 2018, except HIV-positive status awareness and ART coverage among men, which was 82.9% (67.3% in 2013, p<0.001), and 86.9% (69.4% in 2013, p<0.001), respectively. As in 2013, women were more aware of their HIV-positive status in 2018 than men (p<0.001). Participants aged 15 to 29 years did not reach the first 90 (81.6% versus 62.1% in 2013, p<0.001) and the third 90 (88.8% versus 89.0% in 2013, p = 0.97). HIV-positive status awareness among people aged 15 to 29 years was lower than among those 30 to 59 years old (p<0.001); 61.1% (95%CI: 44.4–75.5) of men aged 15–29 knew their HIV-positive status, the lowest among all subpopulations.

Among the 87 participants unaware of their HIV-positive status in 2018, 79 (90.8%) had previously tested for HIV (100% among women and 77.1% among men), and among them 38 (48.1%) were tested less than 1 year prior to the survey. There was no statistical difference with the results of 2013, when 148/275 (53.8%) of unaware previously tested, had a test done during the year prior to the survey (p = 0.370). In 2018, ART coverage among those who knew their status was higher among women than among men (p<0.001). The lowest ART coverage, at 77.3% (95%CI: 55.4–90.3), was observed among men aged 15–29 years.

There was no statistical difference between 2013 and 2018 in the proportion of individuals on treatment who were virally suppressed: 93.1% versus 94.5% (p = 0.28) respectively. In 2018 viral suppression among those on ART was similar among women and men (p = 0.20), and lower among younger adults than older participants (p = 0.001).

## Discussion

The 2018 population-based survey showed a high proportion of viral suppression in all subpopulations of PLHIV, except men aged 15–29 years. Although significantly improved between 2013 and 2018, viral suppression was still below the target in 2018. The majority of virally unsuppressed PLHIV remained women and individuals aged more than 30 years, likely due to the very high prevalence in both groups. Awareness of HIV-positive status and ART coverage among those aware of their status also improved; the first two UNAIDS 90-90-90 targets were achieved overall, as well as among women and older adults.

Given the evidence that PLHIV on ART with viral suppression do not sexually transmit HIV [1–3], our results are encouraging. They suggest that implementation of community-based services and clinical support at primary care can lead to an increase in HIV status awareness and ART coverage, and high viral suppression within a public sector setting in South Africa. The proportion of people on ART with viral suppression remained similar in all age and gender groups between the two surveys, suggesting that increased proportion of HIV-positive individuals virally suppressed was due to an increase in numbers of people aware of their HIV-positive status and on ART. HIV-positive status awareness increased in all groups, with higher increases–albeit to a lower level–in men and young adults compared to women and older people, suggesting that activities targeting young people and men have been successful to some extent. Moreover, the proportion of participants aware of their HIV-positive status and on ART significantly improved between 2013 and 2018 in all groups, with greater improvements in women and youth below 30 years. In 2018, the large majority of PLHIV who knew their HIV status were on ART, particularly women. These findings suggest that implementation of “UTT” in 2016 [13] was successful in this setting. Interventions to increase HIV status awareness (such as door-to-door testing and decentralised testing sites), to improve linkage to care (through follow up of individuals newly diagnosed with HIV and defaulter tracing by community health workers), to improve retention and adherence (such as scale-up of differentiated models of care and adherence counselling) and to improve quality of care through mentoring are likely to have contributed to these positive results [20–22].

However, despite an impressive improvement in viral suppression between the two surveys, some gaps remained. Men and young adults aged 15–29 years had the lowest viral suppression in 2018, similar to what was observed in 2013 [15,23] and elsewhere in South Africa [24,25]. In 2018, only half the men aged 15–29 years were virologically supressed, with the other half remaining at risk of transmitting HIV. HIV-positive status awareness was also low in this group, with one third unaware of their HIV-infection despite the majority having tested for HIV at least once and almost half testing less than 1 year prior to the survey, suggesting possible recent infection. Improved messaging to promote repeated testing in all groups to reduce the interval between infection and testing is recommended in this setting. This would facilitate earlier initiation of treatment and improved viral suppression, especially among men and younger adults.

The second viral load suppression gap identified among young men is suboptimal ART coverage. Identifying specific interventions to increase ART coverage among young men and reduce this demographic disparity is essential in this setting. Finally, as in 2013, young men were the only group not to achieve viral suppression among 90% of those on ART, highlighting the need to improve and/or target adherence counselling for this specific group.

Despite the lower viral suppression among youth and men, the majority of PLHIV with an unsuppressed viral load remained women and older people; half of the unsuppressed were unaware of their HIV-positive status and an increasing proportion of the unsuppressed were on ART. Therefore, targeted approaches to reach women earlier and increased targeted adherence support remain the highest priority to decrease transmission.

### Strengths and limitations

The population-based survey design minimised bias inherent to routinely available data. Our results are generalizable to the entire population of Eshowe/ Mbongolwane, including to HIV-positive individuals who were not previously diagnosed. However, this design does not allow for assessing causality. The participation rate was lower in 2018 compared to 2013, specifically among men. This may have led to underestimation or overestimation of the prevalence and the UNAIDS 90-90-90 targets results if we assume that those absent at the time of the survey, or who refused to participate were more or less likely to be HIV-positive than the general population. Self-reporting bias may also have occurred in both years. For instance, awareness of HIV positivity may have been underestimated among participants who falsely reported not knowing their HIV-positive status. Self-reported ART coverage may have been overestimated or underestimated in 2018, even though it has been previously demonstrated in this setting that there was no major difference between self-reporting and an ARV blood detection test to estimate ART coverage [19].

Progress toward the UN’s ambitious “90-90-90” goal must be continually assessed and shared. This survey’s data may prove particularly informative as a benchmark in HIV progress prior to the arrival of the COVID-19 pandemic, which has had devastating global impacts on HIV care. The six-year interval of our surveys, which use active, population-based evidence rather than limited data extrapolated from selected sites or modelling, allows enough time for HIV and Ministry of Health actors to see the real impact of their health policy choices. Future research and policy, when compared to our results, may provide insight as to how well or poorly these actors were able to assure the continuity of HIV care despite COVID- 19.

## Conclusion

The proportion of HIV-positive individuals virally suppressed improved significantly from 2013 to 2018, in all age and gender groups of PLHIV, mainly due to an increase of HIV-positive status awareness and/or ART coverage. However, a number of youths remained untested, untreated and unsuppressed. Nevertheless, the majority of virally unsuppressed PLHIV were women and older adults. Key challenges remain to further increase viral suppression among all PLHIV. While linkage to ART after HIV testing has dramatically improved, leaving few PLHIV aware of their status untreated, half of the unsuppressed was unaware of their status. Specific strategies are needed to target earlier and frequent testing.

## Supporting information

S1 File (DTA)
