# Correction: Increase in HIV viral suppression in KwaZulu-Natal, South Africa: Community-based cross sectional surveys 2018 and 2013. What remains to be done?

**DOI:** 10.1371/journal.pone.0299408

**Published:** 2024-02-20

**Authors:** Nolwenn Conan, Erica Simons, Menard L. Chihana, Liesbet Ohler, Ellie FordKamara, Mduduzi Mbatha, Gilles vanCutsem, Helena Huerga

There are errors in the Funding and Competing Interests statements. The correct statements are:

**Funding:** This survey was funded by Médecins Sans Frontières (MSF)—Operational Centre Brussels. MSF provided support in the form of salaries for NC, ES, LO, EFK, and GVC. The specific roles of these authors are articulated in the ‘author contributions’ section. The funder financially supported data collection, study procedures, and study coordination, and was involved with study design, data collection, and analysis, but otherwise had no role in decision to publish or preparation of the manuscript.

**Competing Interests:** The authors have read the journal’s policy and have the following competing interests to declare: MSF provided support in the form of salaries for NC, ES, LO, EFK, and GVC. This does not alter our adherence to *PLOS ONE* policies on sharing data and materials. There are no patents, products in development, or marketed products associated with this research to declare.
